# Clonal hematopoiesis of indeterminate potential (CHIP) in cerebromicrovascular aging: implications for vascular contributions to cognitive impairment and dementia (VCID)

**DOI:** 10.1007/s11357-025-01654-1

**Published:** 2025-04-11

**Authors:** Attila Kallai, Anna Ungvari, Dora Csaban, Zoltan Orfi, Andrea Lehoczki, Jozsef Harasztdombi, Andriy Yabluchanskiy, Zoltán Benyó, Ágnes Szappanos, Stefano Tarantini, Farzaneh Sorond, Péter Sótonyi, Hajnalka Andrikovics, Zoltan Ungvari

**Affiliations:** 1 Institute of Preventive Medicine and Public Health, Semmelweis University, Budapest, Hungary; 2 Doctoral College, Health Sciences Division, Semmelweis University, Budapest, Hungary; 3 Laboratory of Molecular Genetics, Central Hospital of Southern Pest, National Institute for Hematology and Infectious Diseases, Budapest, Hungary; 4 Department of Hematology and Stem Cell Transplantation, Central Hospital of Southern Pest, National Institute for Hematology and Infectious Diseases, Budapest, Hungary; 5 Vascular Cognitive Impairment, Neurodegeneration and Healthy Brain Aging Program, Department of Neurosurgery, University of Oklahoma Health Sciences Center, Oklahoma City, OK USA; 6 Stephenson Cancer Center, University of Oklahoma, Oklahoma City, OK USA; 7 Oklahoma Center for Geroscience and Healthy Brain Aging, University of Oklahoma Health Sciences Center, Oklahoma City, OK USA; 8 Department of Health Promotion Sciences, College of Public Health, University of Oklahoma Health Sciences Center, Oklahoma City, OK USA; 9 International Training Program in Geroscience, Doctoral College/Institute of Preventive Medicine and Public Health, Semmelweis University, Budapest, Hungary; 10 Institute of Translational Medicine, Semmelweis University, 1094 Budapest, Hungary; 11 Cerebrovascular and Neurocognitive Disorders Research Group, HUN-REN, Semmelweis University, 1094 Budapest, Hungary; 12 Heart and Vascular Center, Semmelweis University, Budapest, Hungary; 13 Department of Rheumatology and Clinical Immunology, Semmelweis University, Budapest, Hungary; 14 Department of Neurology, Feinberg School of Medicine, Northwestern University, Chicago, USA; 15 Department of Vascular and Endovascular Surgery, Heart and Vascular Centre, Semmelweis University, Budapest, Hungary; 16 Jozsef Fodor Center for Prevention and Healthy Aging, Semmelweis University, Budapest, Hungary

**Keywords:** Cerebral small vessel disease, Vascular cognitive impairment and dementia, VCI, Aging, Endothelial dysfunction, Inflammation, Blood–brain barrier, Microvascular dysfunction, Cerebrovascular aging, Cognitive decline, Neurovascular unit, Stroke, Neuroinflammation, Cerebral microhemorrhages, Microbleed, Oxidative stress, Hematopoietic stem cells, Neurodegeneration, Vascular aging, White matter hyperintensities, Cerebral blood flow, Atherosclerosis, Inflammaging, Neurovascular coupling, Cerebral hypoperfusion, Cognitive dysfunction

## Abstract

Vascular contributions to cognitive impairment and dementia (VCID) represent a major public health challenge in the aging population, with age-related cerebromicrovascular dysfunction playing a critical role in its development. Understanding the mechanisms underlying cerebromicrovascular aging is crucial for devising strategies to mitigate this burden. Among the key hallmarks of aging, genomic instability and genetic heterogeneity have emerged as significant drivers of age-related diseases. Clonal hematopoiesis of indeterminate potential (CHIP) is a prominent manifestation of this instability, characterized by the non-malignant expansion of hematopoietic stem cell clones that harbor somatic mutations. CHIP is well-established as a contributor to atherosclerosis and cardiovascular disease through its promotion of chronic inflammation. Given that aging is also a major risk factor for cerebral small vessel disease (CSVD) and VCID, it is likely that the same aging processes driving large artery atherosclerosis in CHIP carriers also impair small vessels, including the cerebral microvasculature. While the role of CHIP in large vessel disease is well-documented, its specific contributions to cerebrovascular aging and microvascular dysfunction remain poorly understood. This review explores the potential role of CHIP in age-related cerebrovascular pathologies, with a particular focus on its contribution to CSVD. We discuss how CHIP-related mutations can promote inflammation and oxidative stress, potentially leading to endothelial dysfunction, dysregulation of cerebral blood flow (CBF), blood–brain barrier (BBB) disruption, microvascular inflammation, and cerebral microhemorrhages. Given the potential implications for VCID, elucidating these mechanisms is critical for developing targeted therapies aimed at reducing the burden of cognitive decline in aging populations. This review aims to highlight the current knowledge gaps and encourage further research into the intersection of CHIP, CSVD, and cognitive aging.

## Introduction

Vascular contributions to cognitive impairment and dementia (VCID) represent a growing public health challenge in aging populations worldwide [1–6]. VCID, often driven by cerebral small vessel disease (CSVD), is the second most common cause of dementia after Alzheimer’s disease and accounts for a significant proportion of age-related cognitive decline [1–6]. The pathogenesis of CSVD involves a progressive disruption of cerebrovascular function, including microvascular rarefaction and pathological remodeling, impaired cerebral blood flow regulation, blood–brain barrier (BBB) dysfunction, microvascular inflammation, and cerebral microhemorrhages (CMHs) [7–11]. Despite its clinical significance, the molecular and cellular mechanisms contributing to CSVD and cerebromicrovascular dysfunction and their relationship to systemic aging processes remain poorly understood [11]. Understanding these mechanisms is crucial for developing effective strategies to mitigate the burden of VCID and protect cerebrovascular health in aging populations.

Aging is characterized by a progressive decline in cellular and tissue function, driven by a set of conserved biological processes, collectively referred as the hallmarks of aging [12, 13]. These interconnected mechanisms contribute to the deterioration of physiological resilience and the increased susceptibility to age-related diseases. Among these hallmarks, genomic instability and genetic heterogeneity have gained increasing attention due to their widespread impact on various age-related diseases [14–16]. Clonal hematopoiesis of indeterminate potential (CHIP) is an emerging and striking manifestation of genomic instability, characterized by the expansion of hematopoietic stem cell clones carrying somatic mutations in genes such as *DNMT3 A*, *TET2*, and *ASXL1* [14, 17–20]. While CHIP was initially recognized as a premalignant condition [21, 22] with an increased risk of hematologic malignancies, it has since been implicated in non-hematologic diseases, particularly cardiovascular diseases, through its promotion of chronic inflammation and vascular dysfunction [14, 17, 18].

The link between CHIP and atherosclerotic cardiovascular disease (CVD) is well-established [18, 23–35]. CHIP-mutant leukocytes display increased self-renewal, enhanced inflammasome activation [36–39], and elevated secretion of inflammatory cytokines, including IL- 1β and IL- 6 [23, 24, 33, 40–43]. These inflammatory mediators contribute to endothelial dysfunction [44, 45], plaque progression, and heightened thrombotic risk [46–49].

Given the continuum of vascular aging, it is plausible that CHIP may similarly contribute to microvascular pathologies in the brain [9, 10]. Indeed, aging is the most significant risk factor for both atherosclerosis and CSVD, suggesting shared underlying mechanisms that may extend across the macro- and microvasculature [9, 10]. Despite these insights, the potential role of CHIP in cerebrovascular aging, particularly in the context of CSVD and VCID, remains largely unexplored. The inflammatory, oxidative, and matrix-remodeling properties of CHIP-driven myeloid cells could disrupt the delicate balance of cerebral microvascular homeostasis, promoting BBB dysfunction, microvascular inflammation, and CMHs. Furthermore, CHIP may contribute directly to cerebrovascular pathology through infiltration of mutant cells into the brain’s microvasculature and indirectly via systemic inflammatory mediators that impair vascular function.

In this review, we summarize the current understanding of CHIP, with a focus on its established role in CVD and its potential contribution to age-related cerebromicrovascular pathologies. We discuss the mechanistic pathways through which CHIP may promote CSVD, including inflammation, oxidative stress, endothelial dysfunction, and extracellular matrix degradation. Additionally, we outline key knowledge gaps and suggest potential directions for future research, emphasizing the importance of CHIP as a potential biomarker and therapeutic target in the context of aging-related cerebrovascular and cognitive decline.

## CHIP: from pathogenesis to epidemiology

### Overview of CHIP

Clonal hematopoiesis is a broad term referring to somatic pathogenic variants in hematopoietic cells [14]. These clonal alterations typically involve nucleotide changes, such as single nucleotide variants or small insertions and deletions (indels), as well as larger chromosomal abnormalities, often referred to as mosaic chromosomal abnormalities. The condition is commonly described as age-related clonal hematopoiesis (ARCH) due to its increasing prevalence with advancing age [50–58].

CHIP is specifically defined as the clonal expansion of hematopoietic stem cells (HSCs) harboring somatic mutations in leukemia-associated driver genes, most frequently in *DNMT3 A*, *TET2*, and *ASXL1* [14]. These mutations can be detected in circulating white blood cells or bone marrow cells in individuals without laboratory or morphological evidence of an underlying hematologic malignancy [59, 60]. The occurrence of CHIP becomes more prevalent with age and has been strongly linked to a state of chronic, low-grade systemic inflammation [18, 23, 24, 26, 42].

The clinical significance of CHIP has gained increasing recognition, as accumulating evidence indicates that these mutations contribute to a range of age-related diseases beyond hematology [18, 23–35, 61–66]. CHIP has been implicated in cardiovascular diseases [18, 23–35] and metabolic disorders [67] and is increasingly suspected to play a role in neurodegenerative conditions [68], highlighting its broader influence on aging and the progression of chronic diseases. Understanding CHIP as a manifestation of genomic instability associated with aging provides a unique opportunity to explore the connections between hematopoietic dysfunction, systemic inflammation, and age-related cerebrovascular pathologies [14].

### Pathogenesis of CHIP: genetic basis and epidemiological aspects

CHIP is characterized by the accumulation of somatic mutations in HSCs over time, contributing to clonal expansion and aging-related pathologies [24, 33, 50, 62]. The concept that genetic variations accumulate throughout life, first proposed by Hungarian physicist Leo Szilárd in 1959 [69], aligns with modern theories of aging-related genetic drift and cellular heterogeneity [70]. This concept posits that genetic variations progressively accumulate in stem cell genomes, contributing to age-related pathologies [24, 33, 62], including degenerative and malignant conditions [22, 62, 63]. While many somatic genetic variations, often referred to as passenger or bystander mutations, do not affect cellular function, a subset of mutations in key regulatory genes can promote uncontrolled proliferation, apoptosis resistance, and chronic inflammation [33, 51, 54, 56]. These mutations confer a selective advantage to affected HSCs, allowing them to expand clonally without resulting in overt hematologic malignancy, but they do trigger systemic effects, particularly by promoting sterile inflammation.

Recent advances in DNA sequencing technologies (single-cell or clonal lineage sequencing, ultrahigh-depth sequencing) allowed the in-depth investigations of somatic mutational spectra in aging normal hematopoietic, malignant hematopoietic, and other tissue types [71–73]. Studies utilizing ultrahigh-depth sequencing of hematopoietic stem and progenitor cells (HSPCs) demonstrate a steady accumulation of base substitutions throughout life, with a rate of approximately 14–17 mutations per year [74, 75]. Notably, this mutation rate is slower in HSPCs compared to other tissue types, such as the colon or liver [76]. It is estimated that between 50,000 and 200,000 active HSCs exist in a healthy individual [77], resulting in an accumulation of 60 to 240 million mutated bases over an 80-year lifespan. These somatic mutations, although present in relatively low abundance, may drive clonal expansion when they affect critical regulatory genes [58].

Whole-genome sequencing studies of clonally expanded HSCs reveal that the clonal diversity of hematopoiesis diminishes significantly after age 70. While hematopoiesis in younger adults maintains a highly diverse clonal population, elderly individuals exhibit a marked reduction in clonal diversity, with 12 to 18 clones dominating 30 to 60% of total hematopoiesis. Only a fraction of these clonal expansions harbor known driver mutations, suggesting that the impact of clonal hematopoiesis extends beyond the currently identified genetic drivers [75]. This phenomenon of clonal dominance is observed across various tissues, including skin [78], bronchus [79], and esophagus [80, 81], indicating that the accumulation of somatic mutations is ubiquitous in aging [15].

Epidemiologically, CHIP is prevalent among older adults, with initial studies suggesting that 1 to 5% of individuals in their 50 s harbor CHIP mutations. This prevalence rises sharply with age, reaching 10% in individuals in their 60 s and up to 15% to over 60% in individuals aged 85 and older [41, 61, 82–86]. The application of highly sensitive sequencing methods detecting mutations below 2% VAF, such as error-corrected sequencing, has revealed that a much larger proportion of elderly individuals—20 to 95%—carry pathogenic genetic variants associated with hematologic cancers [87–90]. Importantly, the prevalence of CHIP is influenced not only by age but also by genetic background, with certain populations, such as individuals of Hispanic and East Asian ancestry, showing a lower likelihood of CHIP.

CHIP mutations tend to confer a selective advantage in the setting of chronic inflammation. Inflammatory processes drive the expansion of mutant HSCs by creating an environment in which these cells outcompete their wild-type counterparts [91]. This is particularly evident in *TET2* mutations, where clonal expansion is exacerbated in the presence of inflammatory stimuli [92]. In humans, genotoxic stressors, including chemotherapy, radiation [93], and tobacco smoke [94], also promote the expansion of CHIP clones and increase the risk of progression to hematologic malignancies. Chronic inflammation, driven by conditions like infections [95] or autoimmune diseases [96], provides fertile ground for the selection of CHIP clones, further reinforcing the link between CHIP and systemic diseases.

The identification of CHIP-related mutations, their role in clonal hematopoiesis, and the understanding of the lifestyle and environmental factors [86, 97] that promote their expansion offer new insights into the mechanisms of aging. These findings underscore the significance of CHIP as a driver of both cardiovascular and cerebrovascular diseases, particularly in older populations.

### Detection and characterization of CHIP mutations

The detection of CHIP mutations typically relies on next-generation sequencing (NGS) technologies, which can identify somatic mutations in circulating blood cells at a variant allele frequency (VAF) threshold of 2% or greater [98, 99]. This threshold, corresponding to 4% of circulating cells carrying the mutation in one allele, is somewhat arbitrary but has been widely adopted as the baseline for identifying CHIP mutations [100]. Advances in NGS technologies have significantly improved the detection of smaller clonal populations, allowing for identifying low-frequency mutations that were previously undetectable [101]. Studies have demonstrated that CHIP is more prevalent than initially thought, with low-frequency mutations commonly present in healthy individuals. For instance, CHIP variants with a median VAF as low as 0.2% have been found in middle-aged healthy adults, illustrating that even low-level clonal expansions can be detected using modern sequencing methods [88]. The sensitivity of NGS assays, particularly those incorporating error correction or specialized barcoding techniques, has enabled the detection of CHIP mutations at variant allele frequencies well below the traditional 2% threshold. Large-scale whole-genome sequencing from single cell-derived colonies has further suggested that clones with a VAF of more than 1% are nearly universal in individuals over the age of 70 [75]. These advanced technologies have revolutionized our understanding of CHIP’s prevalence, revealing that somatic mutations are far more common in aging populations than previously believed.

The size of the CHIP clone, as determined by the VAF, plays a crucial role in assessing the risk of hematologic malignancies and cardiovascular diseases. Higher VAFs correlate with increased risk, with studies indicating that clones with a VAF above 10% are significantly associated with an increased likelihood of developing hematologic malignancies. For example, early studies using whole-exome sequencing with a detection limit of 3.5–5% VAF reported hazard ratios (HR) between 11.1 and 12.9 for the development of hematologic malignancies [61, 82]. Clones with a VAF above 10% have been associated with a hazard ratio of 49 for hematologic malignancies, underscoring the importance of clone size in disease progression [61]. The use of error-corrected sequencing (ECS) with a detection limit as low as 0.03% has revealed that CHIP mutations with a VAF above 1% are relevant for predicting the future development of acute myeloid leukemia (AML), while clones below this threshold show a weaker association [102]. Importantly, studies have shown that clonal hematopoiesis can precede AML by many years, but the transformation into malignancy depends on both the size of the clone and the number and type of mutations [103, 104].

In addition to hematologic risks, larger CHIP mutant clones also carry significant cardiovascular risks. Studies have shown that individuals with CHIP clones exceeding 10% VAF have substantially higher risks of cardiovascular events compared to those with smaller clones [26, 59]. Recent evidence suggests that even smaller clones, with VAFs in the range of 0.73–1.15%, can influence cardiovascular outcomes. In these cases, individuals with VAFs above this threshold experienced a 5-year mortality rate of 31–32%, compared to 18–19% for those below the threshold, highlighting the potential need to revise current VAF thresholds [105].

While the risk of developing hematologic malignancies among CHIP carriers is estimated at 1–2% per year, the cardiovascular risk is also elevated in those with larger CHIP clones. This growing body of evidence suggests that the thresholds for clinical significance may need to be gene-specific, as the risk associated with CHIP appears to vary based on the size of the clone and the affected gene. As sequencing technologies continue to evolve, more precise guidelines for the detection and characterization of CHIP mutations will be essential for managing the associated risks of hematologic and cardiovascular diseases in aging populations.

### CHIP mutations and their biological mechanisms

The most commonly mutated driver genes in CHIP are *DNMT3 A*, *TET2*, and *ASXL1*—along with less frequent mutations in *JAK2*, *TP53*, and *PPM1D*, play critical roles in epigenetic regulation, immune modulation, and cell cycle control [106, 107]. These mutations lead to clonal expansion, chronic inflammation, increased risks for hematologic malignancies, and cardiovascular diseases. While mutations in *DNMT3 A* and *TET2* disrupt DNA methylation processes, mutations in *ASXL1* affect chromatin remodeling. Additionally, *JAK2* mutations alter cytokine signaling, *TP53* mutations impair DNA repair and apoptosis, and *PPM1D* mutations suppress tumor suppressor functions. Together, these mutations drive clonal growth and contribute to inflammatory states that exacerbate age-related diseases.

Interestingly, CHIP has been observed not only in humans but also in non-human primates and rodents [108, 109]. In a study involving 60 aged rhesus macaques (median age of 25 years), 12 animals exhibited naturally occurring somatic mutations in driver genes commonly mutated in humans, such as *DNMT3 A* and *TET2*. To better understand the pathological roles of these mutations, researchers employed CRISPR-Cas9 gene editing to introduce loss-of-function mutations in these genes into the HSPCs of young adult macaques [109]. Following autologous transplantation, clones with *TET2* mutations demonstrated robust expansion, while *DNMT3 A*-mutated clones exhibited moderate growth [109]. Notably, clones with *TET2* mutations from these macaques displayed a hyperinflammatory gene expression profile. Additionally, mature macrophages derived from these animals showed elevated activity of the NLRP3 inflammasome and increased production of pro-inflammatory cytokines, including IL- 1β and IL- 6 [109]. Similarly, murine models have been instrumental in elucidating the relationship between CHIP and inflammation. Mice engineered to carry mutations in genes such as *TET2* exhibit clonal expansion of mutant HSPCs, leading to increased production of inflammatory cytokines and a heightened inflammatory state. Collectively, these findings across species underscore the conserved nature of CHIP and its association with systemic inflammation. The presence of CH in both non-human primates and mice provides valuable models for studying its pathophysiological consequences and potential therapeutic interventions.

#### *DNMT3 A* mutations

*DNMT3 A* is the most commonly mutated gene in CHIP, accounting for approximately 58.5% of cases [44]. It encodes a DNA methyltransferase responsible for catalyzing DNA methylation at CpG sites, which regulates gene expression patterns necessary for cell differentiation. Most *DNMT3 A* mutations are loss-of-function variants that increase the self-renewal capacity of HSCs [110], maintaining multipotency-associated genes while suppressing differentiation-associated genes [111]. This selective advantage allows clonal expansion, particularly under inflammatory conditions, such as interferon-gamma stimulation [95], [96] by promoting resistance to stress-induced apoptosis and impairing differentiation [110].

Murine models have shown that *DNMT3 A* mutations can lead to increased expression of inflammatory genes like *Cxcl1*, *Cxcl2*, *IL- 6*, and *Ccl5* further exacerbating inflammation [112] following lipopolysaccharide (LPS) exposure. Clinically, patients with *DNMT3 A*-mutant CHIP often display elevated inflammatory markers such as IL- 1β, IL- 6, and macrophage-derived proteins like Ccl3, Ccl4, and resistin [43, 113], contributing to cardiovascular complications, including cardiac hypertrophy, fibrosis, and impaired cardiac function. This inflammatory environment favors the growth of pro-inflammatory clones, increasing the risk of atherosclerosis and other cardiovascular conditions [112, 114]. *DNMT3 A*-mutated macrophages have increased expression of several monocyte–T-cell interaction molecules like CD300LB, CD83, SIGLEC12, and CD58, altering T-cell function [113].

#### *TET2* mutations

*TET2* mutations are the second most frequent in CHIP, accounting for approximately 20% of cases [44]. *TET2* catalyzes the conversion of 5-methylcytosine to 5-hydroxymethylcytosine, thereby promoting DNA demethylation. Mutations in *TET2* lead to hypermethylation, dysregulating key genes involved in HSC self-renewal, expansion, and differentiation [115]. Additionally, TET2’s noncatalytic roles, such as recruiting histone deacetylase 2 (HDAC2) for transcriptional regulation, also support HSPC homeostasis [116]. Furthermore, *TET2* plays a critical role in restraining inflammation, and loss of function mutations lead to increased production of IL- 1β, IL- 6, IL- 18 [94], and IL- 8 [59], mediated through the activation of the NLRP3 inflammasome [114, 117]. TET2 recruits Hdac1/2 (histone deacetylase1/2) to the IL- 6 promoter DNA segment, regulating IL- 6 expression. TET2 dysfunction therefore leads to higher expression of IL- 6, especially in late-stage inflammation, when the inflammatory trigger is already resolved [118]. TET2 deficient mouse model shows that the IL- 6 receptor is overexpressed in this state, further increasing this circle of inflammation [92], but genetic mutation of IL- 6R which caused lower surface expression of the receptor abrogated the negative cardiovascular effects in a human cohort study [119]. These inflammatory cytokines contribute to the development of CVD and insulin resistance, as observed in *TET2*-mutant mice. The crosstalk between intracellular pathways includes AMP-activated kinase (AMPK) phosphorylation of TET2, which stabilizes the complex. Elevated glucose levels inhibit this process by reducing AMPK activity, leading to dysregulated TET2 function, which promotes proinflammatory cytokine production, tumorigenesis [120], and reduced autophagy [121]. TET2 deficiency-related CHIP elevates macrophage-derived IL- 1β in white adipose tissue, exacerbating insulin resistance in obese or aged mice [122], in line with a significant association between TET2 (HR 1.48; CI 1.05–2.08) and type 2 diabetes [123]. The resulting proinflammatory cytokine production may establish a self-perpetuating cycle, driving CHIP progression [124–126]. In the case of atherosclerosis, this pro-inflammatory niche leads to progressive clonal proliferation as TET2 mutants thrive in this microenvironment and inflammatory cytokine production, further destabilizing the plaque [124, 125].

#### *ASXL1* mutations

Mutations in *ASXL1* occur in approximately 8% of CHIP cases [44]. *ASXL1* encodes a chromatin-binding protein that regulates histone modifications and transcription [127]. Loss-of-function mutations in *ASXL1* disrupt chromatin remodeling, leading to aberrant gene expression. These mutations also promote AKT/mTOR signaling, which enhances cell proliferation, even in the presence of DNA damage [128]. Murine models have demonstrated that *ASXL1*-mutant macrophages produce increased levels of IL- 6 and IL- 1β when exposed to lipopolysaccharides, indicating a pro-inflammatory phenotype [129]. This heightened inflammatory response, driven by the AIM2 inflammasome, further exacerbates cardiovascular risk [130].

#### *JAK2* mutations

Although *JAK2* mutations account for approximately 3% of CHIP cases [44], they are associated with a particularly high risk of cardiovascular events and hematologic malignancies [131]. JAK2 transmits intracellular signals downstream of cytokine receptors as it phosphorylates and activates STAT [132] and regulates TET2 in response to cytokines, thereby linking extracellular signals with epigenetic modifications in hematopoiesis [133] The *JAK2* V617 F gain-of-function mutation, commonly associated with myeloproliferative neoplasms (MPNs), significantly increases the risk of thrombotic events, myocardial infarction, deep vein thrombosis, and stroke [134]. Individuals with somatic *JAK2* V617 F-mutant carriers exhibit elevated levels of IL- 18 and downstream increases in IL- 6 production [94]. *JAK2* V617 F augments the production of neutrophils [135]; additionally, studies have shown that this mutation accelerates atherosclerosis in mouse models [136] by activating the AIM2 inflammasome and promoting IL- 1β production [137]. In murine models, it also speeds up heart failure [138] and atherosclerotic plaque development [139], likely due to enhanced activation of β1 and β2 integrins, which increase leukocyte adhesion to the vascular endothelium [140]. Furthermore, *JAK2* V617 F mutations enhance neutrophil extracellular trap (NET) formation that promotes thrombosis [141]. Consequently, this mutation has the strongest association among all CHIP mutations with arterial thrombosis, elevating the risk of myocardial infarction by 12-fold [59]. *JAK2* V617 F mutation is a known risk factor for potentially life-threatening vascular complications with or without meeting the criteria of myeloproliferative neoplasm.

#### *TP53* mutations

*TP53* gene mutations take up 2% of all mutations [44] and encode the tumor suppressor protein (TP53) which regulates cell cycle, apoptosis, and DNA repair as it has immense effects on cell survival and division [142]. Following its activation by oncogene expression, DNA damage, hypoxia, metabolic dysfunction, or replicative stress, it upregulates target genes to restore balance or lead the cell towards apoptosis [143]. In the context of CHIP, *TP53* somatic mutations emerge usually after different chemotherapies [144] or radiotherapy [93]. In therapy-related acute myeloid leukemia (AML), *TP53* mutations are present in about 30% of cases, whereas in de novo AML, only 5–10% of cases harbor this mutation. The presence of mono- or biallelic TP53 alterations is considered a poor prognostic sign, as it is associated with resistance to conventional therapies, higher relapse rates, and significantly worse overall survival, underscoring its role as an indicator of adverse outcomes in both therapy-related and de novo myelodysplasia or AML [145]. Patients post chemotherapy who developed CHIP shown to have a decreased overall survival 7.6 months vs 16.5 months (*p* = 0.02) compared to patients who did not develop CHIP [146]. *TP53* mutations impair the DNA damage response (DDR), allowing clonal cells to survive and expand under genotoxic stress. Additionally, in patients with TP53 mutations, atherosclerosis progression is another issue. Data analysis revealed that *TP53* CHIP is strongly associated with incident peripheral artery and coronary artery diseases [27]. Progression of anthracycline-induced cardiotoxicity in mice through increased neutrophil invasion is another potential mechanism of the long-term detrimental health effect of *TP53* mutant CHIP [147].

#### *PPM1D* mutations

*PPM1D* mutations account for 4% of CHIP cases [44]. PPM1D works hand in hand with the TP53 protein. Activated TP53 induces the expression of *PPM1D* which encodes a phosphatase that dephosphorylates p53, thus leading to downregulation of TP53-mediated apoptosis [148]. *PPM1D* mutations stabilize the protein, leading to suppressed DDR function like p53-mediated apoptosis; therefore, mutant clones can resist apoptosis and outperform normal HSCs especially when exposed to chemo- or radiation therapy [93, 144, 149, 150]. Individuals with *PPM1D*-mutant CHIP have an increased risk of atherosclerotic cardiovascular disease [27].

### Stability and progression of CHIP clones

One of the defining features of CHIP is the relative stability of mutant clones over time [151]. Most CHIP mutations exhibit slow clonal expansion [152], though certain factors such as chronic inflammation [95] and additional somatic mutations can accelerate this process [73]. Studies have demonstrated that the size of the clonal population, often measured by variant allele frequency (VAF), is a critical determinant of disease risk. Larger CHIP clones, particularly those with a VAF above 10%, are associated with a significantly higher risk of developing hematologic malignancies, cardiovascular diseases, and potentially cerebrovascular complications [26, 59].

The term “indeterminate potential” in CHIP reflects the unpredictability of its clinical consequences. For many individuals, clonal hematopoiesis may remain asymptomatic, while for others, it progresses to malignancy. Earlier studies investigating a relatively small number of cases showed that the majority of CHIP mutations appeared to be relatively stable for extended periods, with either no expansion or a gradual increase. These conclusions warrant careful interpretation because these initial studies lacked patients developing hematologic neoplasm at ongoing assessments, used less sensitive methods, or had short observational periods [61], [152], [153]. However, more sensitive sequencing techniques, such as single-molecule molecular inversion probe sequencing (enabling the detection of VAF below 0.1%), have revealed slow, gradual clonal expansion averaging 7% annually (from 0.01 to 31.5%) [154]. Larger clones (VAF > 2%) were prone for expansion with a median doubling period of approximately 7.4 years [155].

Acquiring secondary mutations, particularly stronger leukemia drivers, plays a critical role in clonal expansion and disease progression. Short telomeres, chromosomal instability, and a pro-inflammatory bone marrow microenvironment are believed to foster the expansion of clonal stem cells, further increasing the risk of additional mutations. The annual rate of progression from CHIP to a myeloid neoplasm is estimated to be 0.5–1% [156], but this risk can vary depending on the specific mutation and clone size. For example, mutations in *DNMT3 A* tend to result in relatively stable clones, while mutations in *TET2, ASXL1*, *TP53*, and *JAK2* are associated with a higher rate of expansion and increased risk of progression [73, 89, 155]. There is growing evidence suggesting that hematopoietic stressors, such as chemotherapy [157–159], radiation therapy [160], and inflammation [95], contribute to both the development of CHIP and its progression to hematologic malignancy [93, 97].

The clonal hematopoiesis risk score (CHRS) [161] model has been developed to estimate the risk of myeloid malignancy based on factors such as mutation type, VAF, and clinical parameters like age, cytopenia, red cell distribution width, and mean corpuscular volume in patients with CHIP or clonal cytopenia of undetermined significance (CCUS) [162]. High-risk mutations include those affecting genes involved in splicing (*SRSF2*, *SF3B1*, *ZRSR2*), AML-related genes (*IDH1*, *IDH2*, *FLT3*, and *RUNX1*), *TP53* and *JAK2*. The CHRS stratified CHIP/CCUS patients into low, intermediate, and high-risk groups with certain cumulative incidences for overt hematological neoplasms. CHRS is also useful for predicting other complications related to CH [162]. The model holds significant potential for early intervention strategies aimed at improving survival by preventing both malignant and nonmalignant outcomes in CHIP/CCUS patients [163, 164]. Evidence from studies highlighted that clonal hematopoiesis in the context of peripheral blood cytopenia poses a significant risk of progressing to a myeloid malignancy, thus warranting vigilant observation. Incorporating features of clonal hematopoiesis into predictive models, along with evaluating hematopoietic stressors, could eventually aid in forecasting and preventing hematopoietic malignancies, and further predictive models are necessitated for predicting the cardiovascular risk associated with the given mutational spectra of the patient [165].

CHIP is analogous to other pre-leukemic states, such as monoclonal gammopathy of undetermined significance (MGUS) and monoclonal B-cell lymphocytosis (MBL) [14]. While CHIP is unique in its involvement of hematopoietic stem cells, MGUS and MBL involve the clonal expansion of plasma cells and B lymphocytes, respectively [14]. In both MGUS and MBL, clonal cell populations can remain stable for extended periods but also have a transformation rate of about 1–2% per year, influenced by specific genetic mutations and additional risk factors [166].

In conclusion, while many CHIP clones remain stable, certain factors such as larger clone size, high-risk mutations, and environmental stressors can accelerate clonal expansion and increase the risk of malignancy and cardiovascular complications. Understanding the factors that influence CHIP progression is essential for predicting disease outcomes and developing strategies for early intervention.

### Risk factors and co-morbidities associated with CHIP

While aging is the primary driver of CHIP development [61], several other factors influence its progression [97] and the associated health risks. These risk factors, in combination with CHIP, contribute to a range of comorbidities, from cardiovascular diseases to immune system impairments and metabolic disorders.

#### Age and genetic predisposition

Advanced age is the most significant risk factor for CHIP, with the prevalence rising sharply in older populations [61]. However, genetic predisposition also plays an important role [167, 168]. Certain common germline variants have been shown to increase susceptibility to CHIP, particularly in specific ethnic groups. For instance, individuals of African ancestry exhibit genetic predispositions located in the *TET2* locus that elevate their risk of developing CHIP [94]. Individuals from European ancestry also have an increased likelihood of developing CHIP. Several novel susceptibility loci associated with the development of clonal hematopoiesis in populations of European origin were identified, affecting genes in DNA repair (*PARP1*, *ATM*, *CHEK2*), hematopoietic stem cell homing (*CD164*), or proto-oncogenes (*TCL1 A*, *SETBP1*) besides the previously identified genes involved in chromosome condensation and segregation (*SMC4*) or telomer-maintenance (*TERT*) [169].

#### Environmental and lifestyle factors

Lifestyle choices, including smoking [170] and diet [171], significantly impact CHIP risk. Smoking is associated with *ASXL1* mutations, potentially through the many carcinogenic chemicals in the fumes causing DNA damage in HSCs. Obesity [172] and poor diet quality [171] characterized by high intake of processed foods have been linked to an increased prevalence of CHIP. Conversely, individuals following healthier lifestyles, with regular physical activity and balanced diets, show lower incidences of CHIP, suggesting that modifiable factors can influence its development [173].

#### Chronic inflammation and immune dysfunction

Chronic inflammation creates an environment that promotes the expansion of CHIP-mutant clones, particularly those in *TET2* and *DNMT3 A* [92, 95]. This inflammatory milieu can result from various conditions, including infections [95], autoimmune diseases [96, 174, 175], and potentially age-related systemic inflammation (inflammaging). CHIP exacerbates the inflammatory response, creating a vicious cycle where inflammation fuels clonal expansion, and the expanding clone produces more inflammatory cytokines. Moreover, CHIP has been linked to immune system impairment and worse outcome in some infectious diseases [176, 177].

#### Metabolic disorders and type 2 diabetes

Emerging evidence suggests that CHIP is involved in the pathogenesis of metabolic disorders, particularly type 2 diabetes [123]. Mutations like *TET2* lead to increased inflammation in adipose tissue, driving insulin resistance [122]. Studies in murine models have demonstrated that Tet2-deficient macrophages exacerbate insulin resistance and metabolic dysfunction, particularly in obese or aged animals [122]. The link between CHIP and metabolic disorders highlights the broader systemic effects of the aging and malfunction of the hematopoietic system.

#### Genotoxic stress from chemotherapy and radiation

Individuals exposed to genotoxic agents, such as chemotherapy [157–159] and radiation therapy [160, 178, 179], are at increased risk of CHIP. CHIP expansion-inducing chemotherapies include platina-based [180] drugs, topoisomerase inhibitors [144], and doxorubicin [150]. These treatments, while effective in eliminating cancer cells, can induce mutations in healthy HSCs or potentially lead to the expansion of pre-existing small mutant clones. Such therapy-related CHIP mutations are particularly associated with a higher risk of progression to myeloid malignancies [164] and cardiovascular complications [27].

## CHIP and cardiovascular disease: a focus on large vessel atherosclerosis

The association between CHIP and cardiovascular disease (CVD) has been extensively documented, with a growing body of evidence highlighting its role in promoting atherosclerosis [24, 28, 33, 181–183]. Epidemiological studies consistently demonstrate that individuals with CHIP mutations, particularly in genes like *DNMT3 A*, *TET2*, *ASXL1*, and *JAK2*, have an increased risk of developing atherosclerotic diseases, including coronary artery disease, peripheral artery disease, myocardial infarction, and stroke [26, 27, 31, 35, 56, 59, 171, 183].

Aging in humans is characterized by chronic, low-grade systemic inflammation, termed “inflammaging,” which contributes to the pathogenesis of multiple age-related diseases [184, 185]. CHIP has emerged as an important factor in this process [186], linking hematopoietic somatic mutations to heightened inflammatory responses and vascular dysfunction. Mechanistically, CHIP drives a pro-inflammatory phenotype through heightened activation of the NLRP3 inflammasome [114], resulting in increased production of interleukins such as IL- 1β and IL- 6 and other pro-inflammatory cytokines [44, 94, 136, 181]. These cytokines contribute to a chronic inflammatory state that disrupts vascular homeostasis by impairing endothelial function, promoting the infiltration of monocytes and macrophages into the vessel wall, and accelerating the formation of atherosclerotic plaques [24, 32, 187].

CHIP-mutant leukocytes exhibit increased self-renewal [110], infiltrate the atherosclerotic plaques [188], and demonstrate enhanced secretion of inflammatory cytokines, matrix metalloproteinases (MMPs), and reactive oxygen species [117, 189]. These factors contribute to extracellular matrix degradation, plaque destabilization, and an elevated risk of plaque rupture and thrombotic events. Simultaneously, ROS production exacerbates oxidative stress, further promoting endothelial dysfunction and vascular inflammation, thereby accelerating the progression of atherosclerosis and increasing the risk of cardiovascular events. This persistent, CHIP-driven inflammation not only accelerates the development of atherosclerosis but also underscores CHIP’s broader impact on vascular aging. By fostering vascular dysfunction and promoting pathological remodeling, CHIP emerges as a significant driver of the inflammatory processes underlying age-related cardiovascular disease and potentially cerebrovascular pathology. Understanding these mechanisms provides a foundation for developing targeted therapies that may mitigate CHIP-associated cardiovascular risk, such as anti-inflammatory agents like IL- 1β inhibitors.

### CHIP and stroke

Stroke remains one of the leading causes of morbidity and mortality worldwide, particularly among older individuals, and is a common trigger of VCID [1, 190, 191]. While traditional risk factors such as hypertension, diabetes, and hyperlipidemia have been well established, recent evidence suggests that CHIP may also play a significant role in the pathogenesis of stroke [186, 192]. Several extensive cohort studies have identified CHIP as an independent risk factor for ischemic stroke [35, 61, 186, 192–197]. A recent meta-analysis identified a significant association between CHIP and stroke risk, reporting a 16% increased risk of all strokes (HR: 1.16; 95% CI: 1.05–1.28) [186] and an 18% increased risk of ischemic stroke (HR: 1.18; 95% CI: 1.06–1.33). Notably, individuals with a variant allele frequency (VAF) ≥ 10% exhibited a comparable risk elevation (HR: 1.19; 95% CI: 1.07–1.33).

### CHIP and coronary artery disease

Individuals with CHIP have a higher rate of all-cause mortality, mainly attributable to an increased risk of coronary artery disease [61, 186]. Strong evidence suggests that CHIP carriers face a 1.9-fold higher risk (95% CI, 1.4–2.7) of coronary artery disease compared to non-carriers, along with a 4.0-fold elevated risk (95% CI, 2.4–6.7) for early-onset myocardial infarction [28]. Among individuals without incident coronary heart disease, CHIP carriers exhibited a median coronary artery calcification score that was 3.3 times higher than non-carriers. In those who subsequently developed coronary heart disease, CHIP was associated with a 1.8-fold increase in calcification scores. Notably, patients with a variant allele fraction (VAF) ≥ 10% faced a 12-fold higher risk of elevated coronary artery calcification, whereas individuals with a VAF < 10% showed no significant increase in risk [28]. In a cohort of patients with ST-elevation myocardial infarction (STEMI), 12% had at least one CHIP mutation, which was associated with increased mortality (HR = 1.967, 95% CI, 1.103–3.507, *p* = 0.022) and a higher incidence of major adverse cardiovascular events (HR = 1.833, 95% CI, 1.154–2.912, *p* = 0.010) even after adjusting for confounders [198]. The CANTOS trial provided further support by demonstrating that targeting inflammation, particularly interleukin- 1β (IL- 1β) in a specific patient group, could reduce recurrent cardiovascular events emphasizing the role of inflammation in CHIP-related cardiovascular pathophysiologies [199, 200].

### Other cardiovascular complications linked to CHIP

The presence of common CHIP variants *DNMT3 A* and *TET2* is significantly associated with an increased risk of mortality combined with heart failure hospitalization (HR, 2.1; 95% CI, 1.1–4.0; *p* = 0.02), independent of traditional cardiovascular risk factors [201, 202]. Recently, mutations in the two most commonly affected genes, *DNMT3 A* and *TET2*, were shown to increase mortality at the low-level VAF of 1.15% and 0.73% in a heart failure cohort [203]. Still, larger clones or multiple mutations further increase the risk of carriers [204]. Relatively young patients with HF have a high chance of underlying CHIP mutation [205], and in a meta-analysis, most common CHIP mutations were associated with HF development (HR = 1.25, 95% CI 1.13, 1.38) [206]. Mechanistically, CHIP contributes to heart failure through both accelerated atherosclerosis and an inflammatory phenotype of myocardial-infiltrating immune cells. CHIP-associated mutations drive the upregulation of the NLRP3 inflammasome and IL- 1/IL- 6 signaling pathways, fostering a chronic pro-inflammatory environment within the myocardium [43, 113]. Additionally, elevated levels of IL- 8, CCL3, CCL4, and resistin have been observed in CHIP carriers with heart failure, exacerbating myocardial fibrosis, impairing systolic function, and increasing cardiac stress. This inflammatory milieu promotes adverse ventricular remodeling and contributes to the progression of heart failure, further underscoring the systemic impact of CHIP beyond hematopoiesis. CHIP is also highly prevalent in hypertrophic cardiomyopathy patients and is associated with worse clinical outcomes [207]. Recently, CHIP has also been associated with atrial fibrillation (AF) [65]. The mechanism of action potentially is through atherosclerosis and inflammation promoting heart failure [208], as well as interleukins like IL- 1b, which induces AF [209–211].

Additionally, CHIP, particularly mutations in *DNMT3 A* and *TET2*, is associated with increased mortality following transcatheter aortic valve implantation (TAVI). Patients with these mutations exhibit pro-inflammatory monocyte and T-cell responses, leading to worse outcomes [212]. CHIP carriers undergoing cardiac surgery also show higher rates of major adverse cardiovascular and cerebral events (MACCE) and mortality compared to non-carriers [213]. Furthermore, studies indicate that patients with cardiogenic shock who carry CHIP mutations have worse clinical outcomes [214, 215].

## Potential role of CHIP in cerebromicrovascular and brain pathologies in aging

### Investigating CHIP’s impact on the microvasculature: a new frontier

CSVD is a major contributor to age-related cognitive decline and a common manifestation of VCID [11, 216–218]. It encompasses a spectrum of pathological changes in the small arteries, arterioles, capillaries, and venules of the brain, leading to impaired CBF regulation, BBB dysfunction, chronic ischemia, and neuroinflammation [11, 216]. These alterations increase the risk of stroke, cognitive impairment, and dementia in aging populations. While aging is the most significant risk factor for CSVD [7, 8, 216, 219, 220], emerging evidence suggests that CHIP-driven inflammation and vascular dysfunction may exacerbate microvascular injury [221–224], thereby contributing to CSVD and its cognitive sequelae [35, 225]. Given CHIP’s well-established role in atherosclerosis and large-vessel cardiovascular disease, it is reasonable to hypothesize that similar mechanisms extend to the cerebral microvasculature, potentially promoting small vessel pathology, white matter damage, and cognitive decline.

#### Epidemiology of CSVD and its overlap with CHIP

CSVD is highly prevalent in older adults, with radiological evidence present in the majority of individuals above the age of 65 [9, 216, 226]. It is estimated that CSVD contributes to 30–50% of dementia cases worldwide, underscoring its public health significance [9, 10]. Traditional risk factors such as hypertension, diabetes, smoking, and dyslipidemia are strongly associated with CSVD [9, 217, 220, 227–229], yet a subset of individuals develop severe small vessel pathology despite the absence of these factors, suggesting additional contributors to microvascular dysfunction. Notably, CHIP shares common risk factors with CSVD, including aging [8] and inflammation [7]. While the direct evidence linking CHIP to CSVD remains limited, several lines of indirect evidence suggest a plausible association between CHIP and microvascular dysfunction in the brain. Recent studies have indicated that CHIP is associated with an increased burden of white matter damage, a hallmark of CSVD [192]. In patients with ischemic cerebrovascular disease, CHIP has also been linked to elevated biomarkers of endothelial dysfunction and systemic inflammation, which are known to drive CSVD progression [230]. Given that CHIP is a well-established risk factor for large-vessel atherosclerosis, it is conceivable that similar inflammatory and endothelial mechanisms contribute to microvascular pathology in CSVD. Despite these associations, the lack of direct studies investigating the role of CHIP in CSVD presents a significant gap in our understanding.

#### Histopathological manifestations of CSVD

CSVD is a heterogeneous condition with diverse histopathological manifestations that contribute to progressive microvascular dysfunction and cognitive decline [229, 231]. One of the key pathological features of CSVD is arteriolosclerosis, characterized by thickening of the vessel wall, loss of smooth muscle cells, and luminal narrowing [231, 232]. These structural changes impair blood flow regulation, leading to chronic cerebral hypoperfusion and increasing the risk of ischemic damage in vulnerable brain regions [233–235]. Another hallmark of CSVD is lipohyalinosis, a degenerative process affecting the walls of small arterioles [236, 237]. This condition, often associated with hypertension, involves fibrinoid necrosis and the accumulation of lipid-laden macrophages, ultimately compromising the integrity of the vessel wall and increasing susceptibility to occlusion or rupture. Another distinct but overlapping pathology within CSVD is cerebral amyloid angiopathy (CAA), which is characterized by the deposition of β-amyloid in the walls of small cerebral arteries and arterioles [238–240]. The accumulation of amyloid weakens the vessel wall, making it more susceptible to rupture and increasing the risk of cerebral hemorrhages, particularly lobar hemorrhages in older adults. CAA is commonly observed in individuals with Alzheimer’s disease, further linking CSVD to neurodegenerative processes [238, 240]. Endothelial dysfunction is a fundamental mechanism underlying CSVD [241–245]. It often associates with microvascular rarefaction [246, 247], a reduction in the density of functional small vessels, and increased permeability of the BBB [10, 248]. The disruption of BBB integrity allows inflammatory mediators and circulating toxins to enter the brain parenchyma, exacerbating neuroinflammation and white matter damage [249–252]. In addition to these structural changes, microinfarcts and microhemorrhages [9, 253] are frequently observed in CSVD and contribute significantly to cognitive impairment. Microinfarcts result from the occlusion of small penetrating arterioles, leading to localized ischemic damage [254–260], while microhemorrhages reflect the rupture of fragile capillaries or arterioles, leaving behind hemosiderin deposits [9, 253]. The cumulative burden of these small lesions disrupts neuronal connectivity and accelerates cognitive decline, particularly in the presence of concurrent neurodegenerative diseases. Together, these pathological features of CSVD create a vicious cycle of microvascular dysfunction, chronic inflammation, and progressive neuronal injury, manifesting as VCID. Understanding the cellular and molecular drivers of these processes, including the potential role of CHIP, may provide new insights into strategies for preventing and managing age-related cerebrovascular disease.

#### Diagnosis of CSVD: MRI biomarkers

CSVD is primarily diagnosed using magnetic resonance imaging (MRI), which enables the visualization of characteristic structural and functional changes associated with small vessel pathology [227, 228, 261, 262]. MRI biomarkers provide crucial insights into the extent and severity of CSVD-related brain injury, helping to distinguish its contributions to age-related cognitive decline [261, 262]. The key MRI markers of CSVD include white matter hyperintensities (WMHs), cerebral microbleeds (CMH), lacunar infarcts, perivascular spaces (PVS), and cortical and subcortical atrophy [262, 263].

WMHs appear as regions of increased signal intensity on T2-weighted and fluid-attenuated inversion recovery (FLAIR) imaging, reflecting demyelination, gliosis, and ischemic damage. WMHs are a hallmark of CSVD and correlate with cognitive impairment, particularly executive dysfunction and processing speed deficits [264, 265]. CMHs are small, punctate hemosiderin deposits visible on susceptibility-weighted imaging (SWI) or gradient-recalled echo (GRE) sequences, indicative of microvascular fragility and hemorrhagic damage [9, 253]. The presence of multiple CMHs is associated with an increased risk of cognitive decline and intracerebral hemorrhage [9, 253]. Lacunar infarcts are small ischemic lesions (< 15 mm) affecting the deep gray matter (e.g., basal ganglia, thalamus) or white matter, resulting from occlusion of penetrating arterioles [266]. Also known as Virchow-Robin spaces, PVS are enlarged fluid-filled spaces surrounding small penetrating arterioles that can be visualized on T2-weighted MRI [267–271]. The dilation of PVS is believed to reflect impaired fluid clearance, increased vascular permeability, and microvascular dysfunction. An excessive burden of PVS has been linked to worse cognitive outcomes in aging populations [270]. Additionally, progressive loss of brain volume, particularly in subcortical structures and cortical areas, often accompanies CSVD. While atrophy is traditionally associated with neurodegenerative conditions such as Alzheimer’s disease, increasing evidence suggests that vascular contributions to neurodegeneration exacerbate cortical thinning and white matter atrophy in CSVD-related cognitive decline.

#### Functional cerebromicrovascular contributions to VCID

CSVD disrupts the functional integrity of the neurovascular unit, leading to impaired neurovascular coupling (NVC), dysregulation of CBF, resulting chronic cerebral hypoxia, BBB disruption, related neuroinflammation, and increased susceptibility to neurodegeneration [272].

Several key age-related mechanisms by which CSVD contributes to cognitive impairment include microvascular endothelial dysfunction and reduced NVC [272]. Age-related increases in endothelial oxidative stress and mitochondrial dysfunction lead to decreased bioavailability of NO, vasoconstriction, and chronic cerebral hypoperfusion [273–277]. The failure of small cerebral vessels to adequately dilate in response to metabolic demands (impaired NVC) compromises oxygen and nutrient delivery to neurons, particularly in the subcortical white matter, which is highly sensitive to ischemic injury [272]. CSVD is also associated with increased permeability of the BBB [249–252], allowing circulating inflammatory mediators, immune cells, and neurotoxic molecules to infiltrate the brain parenchyma [248, 261]. BBB leakage contributes to perivascular inflammation, astrocyte activation, and neurovascular uncoupling, exacerbating neuronal damage and cognitive impairment [249–252]. Chronic BBB disruption leads to the activation of microglia and astrocytes, which release pro-inflammatory cytokines such as IL- 1β, IL- 6, and TNF-α, further amplifying neuroinflammation [249–252]. This inflammatory cascade disrupts neuronal homeostasis, accelerates white matter degeneration, and contributes to the development of subcortical dementia. Reduced CBF compromises energy metabolism in vulnerable brain regions, leading to progressive myelin loss, axonal injury, and gliosis [233]. Chronic hypoperfusion, BBB disruption, and neuroinflammation are strongly correlated with the severity of WMHs, which are associated with executive dysfunction, processing speed deficits, and gait disturbances [278]. Additionally, CSVD frequently coexists with Alzheimer’s disease [279, 280], and accumulating evidence suggests that cerebrovascular dysfunction may exacerbate amyloid-β and tau pathology [250–252, 281, 282].

#### Continuum of vascular aging and the potential overlap in mechanisms between large and small vessel diseases

Vascular aging represents a continuum of pathological changes from large arteries to the microvasculature, driving atherosclerosis and CSVD in parallel [9, 10, 283]. Traditionally viewed as distinct entities, atherosclerosis, which primarily affects large arteries, and CSVD, which involves the cerebral microvasculature, share overlapping pathophysiological mechanisms [9, 10]. Both conditions are age-related, progressive, and involve shared mechanisms of aging, such as increased reactive oxygen species (ROS) production, impaired nitric oxide availability, cellular NAD + depletion, SIRT1 dysregulation, and the promotion of inflammatory processes, leading to endothelial dysfunction [9, 10]. Vascular risk factors that accelerate aging and atherogenesis in large arteries also promote microvascular aging and neurovascular dysfunction, underscoring the systemic nature of vascular aging and the interconnected nature of vascular health [9, 10].

Endothelial dysfunction and oxidative stress are fundamental drivers of both atherosclerosis and CSVD in older adults [9, 10]. In the large arteries, dysfunctional endothelial cells contribute to atherogenesis by promoting monocyte adhesion, platelet aggregation, and vascular inflammation [284]. In the cerebral microcirculation, endothelial dysfunction compromises NVC, leading to impaired CBF regulation, BBB permeability, and an increased risk of ischemic damage [272, 283]. Reduced bioavailability of NO, a key regulator of vascular homeostasis, is a central feature of both diseases [9, 10]. Mitochondrial dysfunction and increased mitochondria-derived ROS production play a crucial role in age-related endothelial dysfunction and vascular inflammation both in large vessels and at the level of the microcirculation [184, 275, 285, 286]. Oxidative stress-induced DNA damage triggers cellular senescence in endothelial cells both in large vessels and cerebral microvessels [184, 185]. Senescent cells exhibit a pro-inflammatory senescence-associated secretory phenotype (SASP), secreting cytokines and matrix-degrading enzymes that exacerbate plaque instability in large arteries and promote microvascular rarefaction and white matter damage in the brain [184, 185, 287].

Chronic low-grade inflammation is a hallmark of both large and small vessel diseases, contributing to vascular dysfunction, fibrosis, and impaired vascular repair mechanisms [184, 185]. In atherosclerosis, inflammatory cytokines drive plaque formation and instability [46, 184, 185, 288]. Similarly, in CSVD, systemic inflammation promotes BBB disruption and perivascular inflammation, accelerating white matter damage and neurodegeneration [10, 289]. Notably, CHIP has emerged as a potential unifying factor linking systemic inflammation to vascular pathologies [24, 222, 224]. CHIP carriers exhibit increased production of inflammatory cytokines [290], which may exacerbate endothelial dysfunction and vascular aging across the entire vascular tree. This suggests that CHIP may contribute to both atherosclerotic and microvascular disease through similar inflammatory pathways.

The BBB plays a critical role in maintaining cerebral homeostasis, and its dysfunction is a common feature of CSVD [250–252]. Increasing evidence suggests that systemic vascular dysfunction, including atherosclerosis, contributes to BBB disruption [10]. Large artery disease can lead to impaired cerebrovascular autoregulation, resulting in chronic hypoperfusion, oxidative stress, and inflammation, all of which compromise BBB integrity [10]. Furthermore, increased permeability of the BBB in CSVD allows for the infiltration of circulating inflammatory mediators and immune cells, exacerbating neuroinflammation and neuronal injury. The breakdown of the BBB also impairs the clearance of toxic proteins, potentially linking vascular pathology to neurodegenerative diseases [250–252].

Arterial stiffness, a key feature of large vessel aging, contributes to increased pulsatile flow in the cerebral microcirculation, which may accelerate microvascular damage and increase the risk of microhemorrhages [9]. In individuals with large artery atherosclerosis, excessive pulsatility from stiffened arteries may be transmitted to the fragile cerebral small vessels, promoting microvascular rupture, BBB breakdown, and microhemorrhage formation [9]. Recent studies suggest that individuals with significant carotid atherosclerosis exhibit greater WMHs and CMHs, suggesting a direct link between macrovascular disease and microvascular pathology [9, 10].

These findings highlight that vascular aging is not an isolated phenomenon affecting distinct vessel types but rather a continuum of systemic dysfunction spanning both large arteries and the microvasculature. Given its well-established role in promoting vascular inflammation, endothelial dysfunction, and immune activation, CHIP emerges as a key factor that may contribute to many of the aforementioned mechanisms, accelerating the pathogenesis of both large and small vessel disease.

### Indirect evidence implicating CHIP in CSVD and age-related microvascular dysfunction

Although CHIP’s role in large-vessel CVD is well established, its impact on the cerebral microvasculature remains an emerging area of research. Several lines of indirect evidence suggest that CHIP may contribute to the development and progression of CSVD, particularly through mechanisms such as chronic systemic inflammation, generalized endothelial dysfunction, dysregulation of cerebral blood flow, BBB disruption, and microvascular inflammation. While direct studies linking CHIP to CSVD are limited, evidence from cardiovascular and cerebrovascular research strongly supports a plausible connection.

#### Endothelial dysfunction: a potential link between CHIP and CSVD

Endothelial dysfunction is a central feature of both large vessel diseases and microvascular aging, contributing to increased vascular stiffness, impaired autoregulation, and heightened susceptibility to ischemic and hemorrhagic events [9, 10]. The cerebral microvasculature relies on intact endothelial function to maintain CBF, regulate NVC, and preserve BBB integrity. Disruption of these processes is a major driver of CSVD and VCID.

A growing body of research indicates that CHIP mutations contribute to endothelial dysfunction and endothelial injury, at least in part, by promoting systemic inflammation [94, 230, 291, 292]. CHIP carriers exhibit elevated levels of pro-inflammatory cytokines, including IL- 1β, IL- 6, and TNF-α, which can impair endothelial function both directly and indirectly [293–303]. These cytokines have been shown to increase endothelial permeability, promote oxidative stress, and reduce NO bioavailability, leading to widespread vascular dysfunction [296, 304, 305]. CHIP mutations, particularly in *TET2*, *DNMT3 A*, *ASXL1*, and *JAK2* genes, promote a chronic inflammatory state, characterized by increased IL- 6 and IL- 1β production by mutant immune cells. These cytokines induce endothelial activation, upregulating adhesion molecules such as VCAM- 1 and ICAM- 1 and facilitating the recruitment of immune cells to the endothelium [306–312]. The resulting inflammatory cascade leads to endothelial activation, a precursor to vascular dysfunction in both large and small vessel diseases. CHIP-related systemic inflammation is likely also a major driver of ROS production, which exacerbates endothelial damage and accelerates vascular aging [296, 311]. Increased ROS generation reduces NO bioavailability and impairs vasodilation and blood flow regulation. This oxidative stress-induced endothelial dysfunction is a known contributor to both atherosclerosis and CSVD. Although direct studies on CHIP and cerebrovascular endothelial function are limited, several observations support this link. Given that CHIP contributes to atherosclerosis and endothelial dysfunction in the coronary and peripheral arteries, it is likely that similar mechanisms extend to the cerebral microvasculature [9, 10].

Importantly, meta-analyses have identified a significant association between CHIP and stroke risk [35, 192–194, 197]. CHIP carriers with higher variant allele frequency (VAF ≥ 10%) exhibit an increased risk of small vessel stroke, suggesting that CHIP mutations may contribute to microvascular pathology in addition to large-vessel atherosclerosis. Second, elevated circulating levels of inflammatory mediators, known to be upregulated in CHIP carriers, correlate with WMH burden, a key MRI marker of CSVD [7, 289, 313–315]. These inflammatory markers have been linked to BBB dysfunction and microvascular damage, suggesting a potential mechanistic overlap between CHIP-related inflammation and CSVD. Third, the endothelial dysfunction observed in CSVD shares many features with macrovascular atherosclerosis, including impaired NO signaling, oxidative stress, and chronic inflammation, all of which are promoted by CHIP. Taken together, while direct evidence linking CHIP to CSVD remains limited, accumulating indirect evidence supports the hypothesis that CHIP mutations contribute to cerebrovascular endothelial dysfunction, neurovascular uncoupling, and chronic inflammation, ultimately promoting microvascular damage and cognitive decline. Future research should aim to clarify the specific molecular pathways by which CHIP influences the cerebral microvasculature.

#### BBB disruption

The BBB plays a critical role in maintaining cerebral homeostasis by tightly regulating the exchange of substances between the bloodstream and the brain parenchyma [250–252]. This selective permeability protects the central nervous system (CNS) from circulating toxins, inflammatory mediators, and immune cell infiltration. Disruption of the BBB is a hallmark of CSVD, VCID, and Alzheimer’s disease, leading to increased permeability and allowing harmful substances to enter the brain [250–252]. BBB dysfunction has been directly implicated in the pathogenesis of white matter damage in CSVD, contributing to chronic neuroinflammation, neuronal injury, and cognitive decline [250–252].

While BBB disruption is well characterized in aging and cerebrovascular disease [250–252], emerging indirect evidence suggests that CHIP may exacerbate BBB dysfunction through systemic inflammation, oxidative stress, and endothelial activation [250–252]. Many of the same inflammatory pathways that drive large-vessel atherosclerosis in CHIP carriers are also implicated in cerebral microvascular endothelial dysfunction and BBB permeability [316–319]. One of the primary mechanisms by which CHIP may influence BBB integrity is through its pro-inflammatory effects on endothelial cells. CHIP carriers exhibit systemic low-grade inflammation, with elevated circulating levels of pro-inflammatory cytokines such as IL- 1β, IL- 6, and IL- 8 [59, 94]. These cytokines can have profound effects on BBB function [320–326] by disrupting endothelial tight junctions. The BBB relies on tight junction proteins such as occludin, claudins, and junctional adhesion molecules to maintain its structural integrity and limit permeability. CHIP-related chronic inflammation may downregulate or phosphorylate these proteins, leading to weakened intercellular junctions and increased BBB leakage [317, 327]. Further, increased endothelial activation in CHIP carriers may result in the upregulation of adhesion molecules, which likely facilitate immune cell recruitment and extravasation across the BBB. Even in the absence of direct CHIP-mutant cell infiltration into the brain, inflammatory cytokines can cross the BBB, leading to the activation of microglia [328, 329] and astrocytes [330] and perpetuating neuroinflammation. These effects mirror those observed in CSVD, where BBB disruption leads to perivascular inflammation, white matter lesions, and increased vulnerability to ischemia [10, 247, 331, 332]. Given that CHIP is a strong promoter of systemic vascular inflammation, it is likely that CHIP carriers experience accelerated cerebrovascular damage, making BBB integrity a key point of investigation in the CHIP-CSVD link. CHIP-associated systemic inflammation is likely also associated with excessive production of ROS, which further compromises BBB function and promotes neurovascular injury. CHIP mutations have been linked to increased oxidative stress via multiple mechanisms [293, 333, 334]. ROS-mediated BBB damage may occur via lipid peroxidation and endothelial membrane damage, oxidative stress-induced degradation of basal membrane and tight junction proteins [335], activation of the NF-κB pathway [336], and induction and activation of matrix metalloproteinases (MMPs) [337, 338]. CHIP-driven inflammation and oxidative stress likely work synergistically to accelerate BBB breakdown, leading to enhanced immune cell infiltration, vascular leakage, and chronic neuroinflammation—a well-recognized pathway in CSVD-related cognitive impairment.

#### Endothelial activation, transmigration of leukocytes, and microvascular inflammation

A likely consequence of CHIP-related inflammation is the activation of endothelial cells, characterized by increased expression of adhesion molecules [339, 340]. Endothelial activation facilitates immune cell recruitment and transmigration across the BBB [341, 342], leading to perivascular inflammation and microvascular damage [343] and an increase in vascular permeability [344, 345]. Emerging evidence suggests that CHIP-mutant cells themselves can extravasate into the brain parenchym [68]. Once inside the CNS, transmigrated monocytes and neutrophils likely can further exacerbate neuroinflammation by releasing ROS, MMPs, and pro-inflammatory cytokines, contributing to BBB disruption, synaptic dysfunction, and white matter injury—all hallmarks of CSVD and VCID.

Microglia, the resident immune cells of the brain, play a critical role in maintaining neuronal homeostasis and responding to injury. Microglia typically maintain their population through self-renewal and also through recruitment from blood-derived monocytes. Under pathological conditions, such as chronic inflammation and BBB dysfunction, infiltrating hematopoietic-derived monocytes can engraft into the CNS and adopt a microglia-like phenotype [346–350]. Some evidence suggests that CHIP-mutant monocytes may undergo this transformation, as studies have identified a correlation between CHIP mutation burden in peripheral blood and the presence of CHIP-associated cells in the brain [351]. If CHIP-mutant immune cells do integrate into the CNS as microglia-like cells, they may adopt a pro-inflammatory phenotype, perpetuating chronic neuroinflammation and microvascular injury. This could have profound implications for CSVD progression, CMHs, and cognitive decline.

#### Increased microvascular fragility and the role of CHIP-related inflammation

Microvascular fragility is a defining feature of CSVD and plays a critical role in the development of CMHs [253]. While aging alone contributes to the structural weakening of cerebral vessels [253, 352–354], CHIP-related inflammation may serve as an additional driver of microvascular fragility through chronic immune activation, oxidative stress, and dysregulated ECM remodeling. The systemic and localized pro-inflammatory environment induced by CHIP mutations could contribute to excessive MMP activation, disrupting endothelial integrity and predisposing the cerebral microvasculature to hemorrhagic events. MMPs play a key role in ECM degradation, weakening the basement membrane, and increasing vascular permeability [253, 354, 355]. While direct evidence of CHIP-mutant cells actively secreting MMPs in the brain remains scarce, their role in other vascular compartments—including atherosclerosis and myocardial fibrosis—suggests that similar mechanisms may be at play in the cerebral microcirculation. Another potential contributor to CHIP-induced microvascular fragility is oxidative stress, which is strongly linked to CHIP through the upregulation of ROS-generating pathways [253, 354, 355]. This cascade of inflammation, ECM breakdown, and oxidative injury may amplify the susceptibility of small cerebral vessels to rupture, leading to CMHs, BBB disruption, and the progression of CSVD-related cognitive impairment [253]. We propose that the interplay between CHIP-induced chronic inflammation, oxidative stress, and MMP activation establishes a self-perpetuating cycle of vascular injury. This cascade may accelerate the age-related decline of the cerebral microcirculation, predisposing CHIP carriers to more severe cerebrovascular damage, increased burden of CMHs, and heightened expression of VCID. Future studies should investigate whether CHIP mutations correlate with specific patterns of microvascular hemorrhages or white matter injury in aging populations, particularly in individuals with high CHIP variant allele frequencies.

## Clinical implications and future directions: what to do with CHIP?

The growing recognition of CHIP as a significant risk factor for both cardiovascular and cerebrovascular disease raises important clinical and research questions. While CHIP is often detected incidentally, its strong associations with atherosclerosis, coronary artery disease, stroke, and possibly neurodegeneration suggest that it could serve as both a biomarker for risk stratification and a potential therapeutic target. However, integrating CHIP into clinical decision-making remains challenging due to the lack of established guidelines and definitive treatment strategies.

One of the key clinical implications of CHIP is its potential role in refining risk stratification for cardiovascular and cerebrovascular events. Individuals with CHIP, particularly those with a high variant allele frequency (VAF ≥ 10%) or specific mutations such as JAK2, are at a heightened risk for myocardial infarction, stroke, and heart failure [59]. Emerging evidence suggests that these patients may require more intensive monitoring, particularly in those who present with unexplained or premature cardiovascular disease. Current risk models may underestimate the impact of CHIP-driven inflammation and endothelial dysfunction, highlighting the need to incorporate CHIP status into clinical assessments. In high-risk individuals, additional vascular evaluations such as coronary artery calcification (CAC) scoring, carotid intima-media thickness measurements, or MRI assessment of cerebral small vessel disease could provide valuable prognostic information.

Given that systemic inflammation is a primary mechanism linking CHIP to vascular disease, targeting inflammatory pathways has emerged as a promising therapeutic strategy. Several classes of anti-inflammatory therapies are being explored for their potential benefits in CHIP carriers. IL- 1β inhibition, particularly with canakinumab, has already demonstrated efficacy in reducing cardiovascular events in patients with elevated C-reactive protein, even in the absence of hyperlipidemia [356]. This suggests that CHIP carriers, who frequently exhibit chronic inflammation, might benefit from similar interventions. Likewise, IL- 6 inhibitors, such as tocilizumab and ziltivekimab, could mitigate the inflammatory burden associated with CHIP-related vascular dysfunction [139, 356]. Another emerging approach involves targeting the NLRP3 inflammasome, a key regulator of inflammatory responses that is activated in CHIP-driven vascular pathology [357].

Beyond inflammation, CHIP’s impact on thrombosis risk [47, 291, 358, 359] has raised the question of whether CHIP-positive individuals require modifications to their antiplatelet or anticoagulant regimens. Patients carrying *JAK2* mutations, in particular, exhibit a pro-thrombotic phenotype, suggesting that thromboprophylaxis might be warranted in these individuals. In patients with atrial fibrillation, CHIP has been associated with an increased risk of stroke, raising the possibility that earlier or more intensive anticoagulation strategies could be beneficial. While no formal guidelines exist for CHIP-based adjustments to antithrombotic therapy, these considerations highlight the need for future clinical trials to determine whether CHIP status should influence anticoagulation decisions.

The implications of CHIP extend beyond CVD to cerebrovascular pathology, particularly in the context of CSVD and vascular contributions to cognitive impairment. Given the association between CHIP and stroke, as well as indirect evidence linking CHIP to endothelial dysfunction, it is possible that CHIP plays a role in the development of cognitive decline. Future research should explore whether CHIP carriers, particularly those with high VAF, exhibit an accelerated trajectory of neurovascular aging and dementia risk. If CHIP is indeed a driver of VCID, early intervention with anti-inflammatory or neuroprotective therapies could be an additional strategy for preserving cognitive function in aging populations.

While the evidence linking CHIP to vascular disease is compelling, routine screening for CHIP in asymptomatic individuals remains controversial. At present, there is no consensus on whether CHIP testing should be incorporated into standard clinical practice. One argument against widespread screening is the current lack of CHIP-specific therapies, raising concerns that identifying CHIP could increase patient anxiety without offering clear clinical benefits. However, targeted screening in select populations—such as individuals with unexplained or early-onset cardiovascular disease, recurrent strokes, or chemotherapy-induced CHIP [147]—could provide valuable prognostic information and guide personalized preventive strategies.

Looking ahead, several critical research questions remain unanswered. It is still unclear whether CHIP is a direct driver of cerebrovascular pathology or whether its effects are mediated entirely through systemic inflammation. Further studies are needed to determine whether specific CHIP mutations carry a higher risk of stroke, CSVD, or cognitive decline and whether targeted interventions can mitigate these risks. Additionally, understanding how CHIP-mutant immune cells can infiltrate the brain and contribute directly to neuroinflammation is a crucial area of investigation.

In conclusion, CHIP is emerging as an important factor linking hematopoietic aging to vascular dysfunction, inflammation, and disease. While its role in cardiovascular pathology is well established, its impact on cerebrovascular health remains an area of active research. Integrating CHIP into risk stratification models, refining therapeutic strategies, and developing targeted interventions will be essential steps in translating these findings into clinical practice. Future research should focus on clarifying the mechanisms by which CHIP influences vascular aging, identifying high-risk individuals, and evaluating whether CHIP-directed therapies can improve long-term cardiovascular and cognitive outcomes.

## Data Availability

N/A.
