# Correction to: Clonal hematopoiesis of indeterminate potential (CHIP) in cerebromicrovascular aging: implications for vascular contributions to cognitive impairment and dementia (VCID)

**DOI:** 10.1007/s11357-026-02210-1

**Published:** 2026-03-19

**Authors:** Attila Kallai, Anna Ungvari, Dora Csaban, Zoltan Orfi, Andrea Lehoczki, Jozsef Harasztdombi, Andriy Yabluchanskiy, Zoltán Benyó, Ágnes Szappanos, Stefano Tarantini, Farzaneh Sorond, Péter Sótonyi, Hajnalka Andrikovics, Zoltan Ungvari

**Affiliations:** 1 Institute of Preventive Medicine and Public Health, Semmelweis University, Budapest, Hungary; 2 Doctoral College, Health Sciences Division, Semmelweis University, Budapest, Hungary; 3 Jozsef Fodor Center for Prevention and Healthy Aging, Semmelweis University, Budapest, Hungary; 4 Laboratory of Molecular Genetics, Central Hospital of Southern Pest, National Institute for Hematology and Infectious Diseases, Budapest, Hungary; 5 Department of Hematology and Stem Cell Transplantation, Central Hospital of Southern Pest, National Institute for Hematology and Infectious Diseases, Budapest, Hungary; 6 Vascular Cognitive Impairment, Neurodegeneration and Healthy Brain Aging Program, Department of Neurosurgery, University of Oklahoma Health Sciences Center, Oklahoma City, OK USA; 7 Stephenson Cancer Center, University of Oklahoma, Oklahoma City, OK USA; 8 Oklahoma Center for Geroscience and Healthy Brain Aging, University of Oklahoma Health Sciences Center, Oklahoma City, OK USA; 9 Department of Health Promotion Sciences, College of Public Health, University of Oklahoma Health Sciences Center, Oklahoma City, OK USA; 10 International Training Program in Geroscience, Doctoral College/Institute of Preventive Medicine and Public Health, Semmelweis University, Budapest, Hungary; 11 Institute of Translational Medicine, Semmelweis University, 1094 Budapest, Hungary; 12 Cerebrovascular and Neurocognitive Disorders Research Group, HUN‑REN, Semmelweis University, 1094 Budapest, Hungary; 13 Heart and Vascular Center, Semmelweis University, Budapest, Hungary; 14 Department of Rheumatology and Clinical Immunology, Semmelweis University, Budapest, Hungary; 15 Department of Neurology, Feinberg School of Medicine, Northwestern University, Chicago, USA; 16 Department of Vascular and Endovascular Surgery, Heart and Vascular Centre, Semmelweis University, Budapest, Hungary

**Correction to: GeroScience (2025) 47:2739–2775**

10.1007/s11357-025-01654-1

The Acknowledgements section was missing from this article and should have read “Project no. 151053 has been implemented with the support provided by the Ministry of Culture and Innovation of Hungary from the National Research, Development and Innovation Fund, financed under the National Research Excellence Programme ADVANCED_24 funding scheme. This work was also supported by the 2015-1.2.1.-HU-RIZONT-2-25-00016 (INNOBRAIN) and the Mission-driven National Cardiovascular Laboratory Program (2026.4.1.1.) from the source of the Hungarian National Research, Development and Innovation Fund and by the EKÖP-2025-323 New National Excellence Program of the Ministry for Culture and Innovation, through the National Research, Development and Innovation Fund.”.

